# Radiological Diagnosis and Emergency Management of Aortic Dissection in a Patient With a Fused Bicuspid Aortic Valve: A Case Study

**DOI:** 10.7759/cureus.73426

**Published:** 2024-11-11

**Authors:** Thshreen S Alfazza'a, Suleiman N Almashaqbeh, Diyaa A Saleh, Samer H Ibrahim, Noof Y Alnemrat

**Affiliations:** 1 Emergency, Princess Iman/Maadi Hospital, Deir Alla District, JOR; 2 Internal Medicine, New Zarqa Governmental Hospital, Zarqa, JOR; 3 General Practice, New Zarqa Governmental Hospital, Zarqa, JOR; 4 Internal Medicine, Royal Medical Services, Irbid, JOR; 5 Emergency, Princess Basma Hospital, Irbid, JOR

**Keywords:** atypical chest pain, bicuspid aortic valve, cardiac tamponade, epigastric pain, pericardial effusion, type a aortic dissection

## Abstract

This report presents a case of an acute Type A aortic dissection in a young patient with atypical symptoms, highlighting the importance of prompt radiology-aided diagnosis and intervention. A 29-year-old male with no significant medical history presented with right upper quadrant and epigastric pain, along with leg numbness. Extensive imaging revealed an ascending aortic dissection with a 5.1 cm aneurysm and moderate-to-severe pericardial effusion. After initial stabilization, an emergency Bentall procedure with mechanical valve replacement was performed. It emphasizes the importance of considering aortic dissection in young patients with atypical symptoms, as it can mimic other conditions, complicating timely diagnosis and management. The postoperative course was uneventful, and the patient stabilized in the intensive care unit (ICU). Early recognition and rapid surgical intervention are crucial in managing atypical aortic dissection cases, especially in younger patients with minimal risk factors.

## Introduction

Valvular heart diseases, including those related to bicuspid aortic valves, are increasingly recognized for their global health impact. The bicuspid aortic valve is the most common congenital cardiac condition, affecting around 1-2% of the population [1-2]. Valvular diseases are also known to cause atypical symptoms in various patients, making it crucial to consider these conditions in acute settings.

Aortic dissection is a life-threatening condition that requires immediate medical attention. It typically occurs in older adults, particularly those with underlying risk factors like hypertension or connective tissue disorders. However, patients with bicuspid aortic valve may experience dissection much earlier, often in their 20s or 30s. 

In these patients, dissection tends to present at an earlier age and is commonly associated with conditions like aortic aneurysms. Early detection and intervention are vital, as is the role of imaging in monitoring individuals at higher risk for aortic disease. Patients with bicuspid aortic valves who develop aneurysms are at increased risk for aortic dissection, which necessitates careful monitoring and regular assessments.

Here, we describe a case of acute Type A aortic dissection in a 29-year-old male who presented with atypical symptoms. This case underscores the importance of maintaining a high index of suspicion to ensure early diagnosis and prompt management.

## Case presentation

A 29-year-old male presented to the Emergency Department on April 12, 2024, with sudden, sharp pain in the right upper quadrant (RUQ) and epigastric region for six hours, accompanied by nausea but without vomiting or other gastrointestinal symptoms. He had no recent trauma or significant medical history other than smoking. No family history of hypertension, connective tissue disorders, or cardiovascular disease was noted.

On initial examination, his vital signs were blood pressure (BP) 148/92 mmHg, mean arterial pressure (MAP 111 mmHg), heart rate 100 bpm, respiratory rate 18 bpm, temperature 37°C, and oxygen saturation of 97% on room air. He was alert and oriented but had visible discomfort. A cardiovascular exam showed normal heart sounds without murmurs, jugular venous distension (JVD), or edema. Lung sounds were clear bilaterally. Abdominal examination revealed tenderness in the RUQ and epigastric regions without rebound tenderness, guarding, or rigidity. Neurovascular assessment identified decreased sensation in both lower extremities but preserved motor strength; distal pulses were present but weaker in the lower extremities, with a delayed capillary refill. Initial investigations, including complete blood count (CBC), basic metabolic panel (BMP), liver function tests (LFTs), coagulation profile, troponin, and lactate, were within normal limits. Both chest X-ray and abdominal ultrasound revealed no abnormalities.

**Figure 1:**
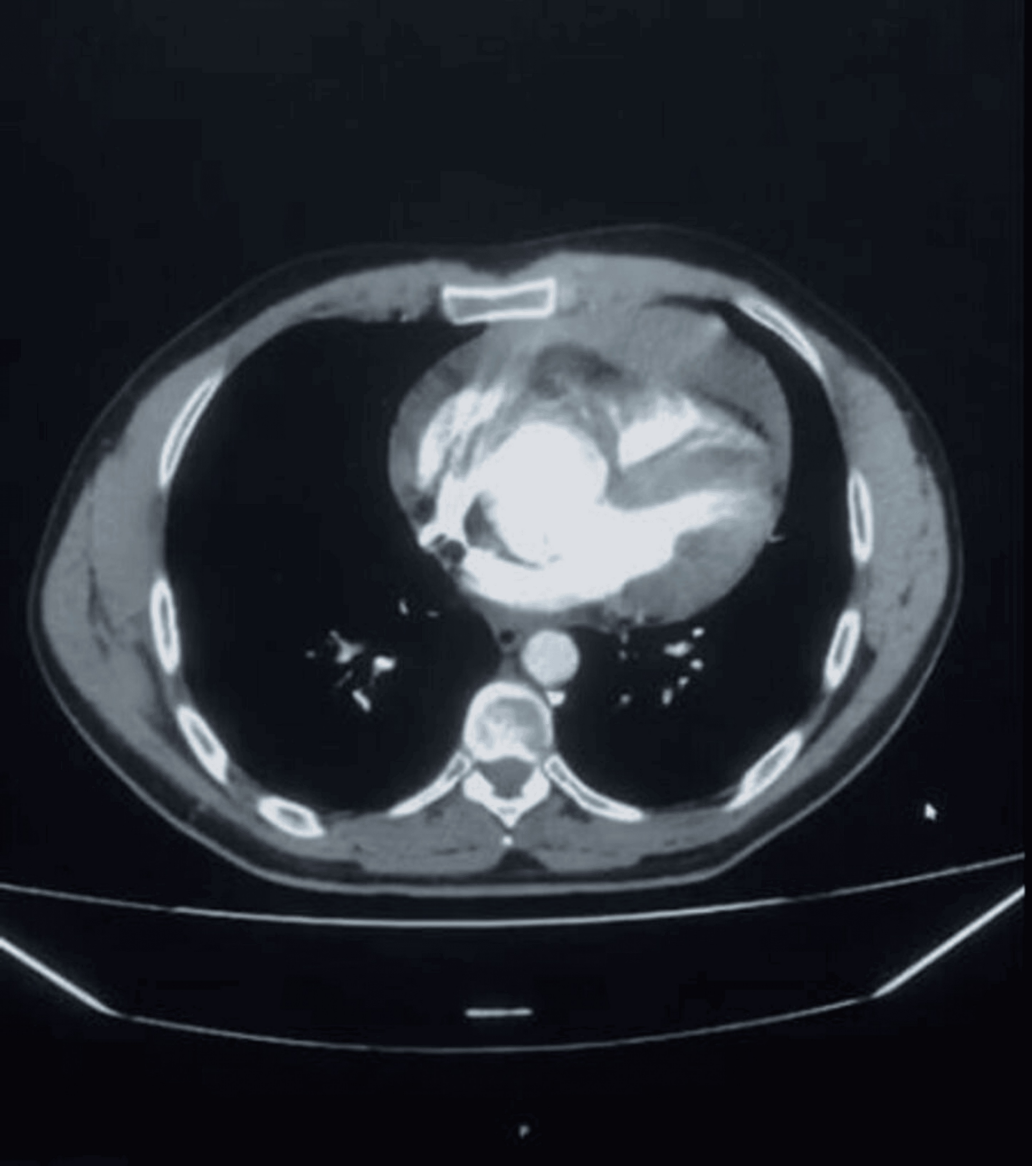
CT with contrast showing the aortic dissection with dilation of the root.

**Figure 2:**
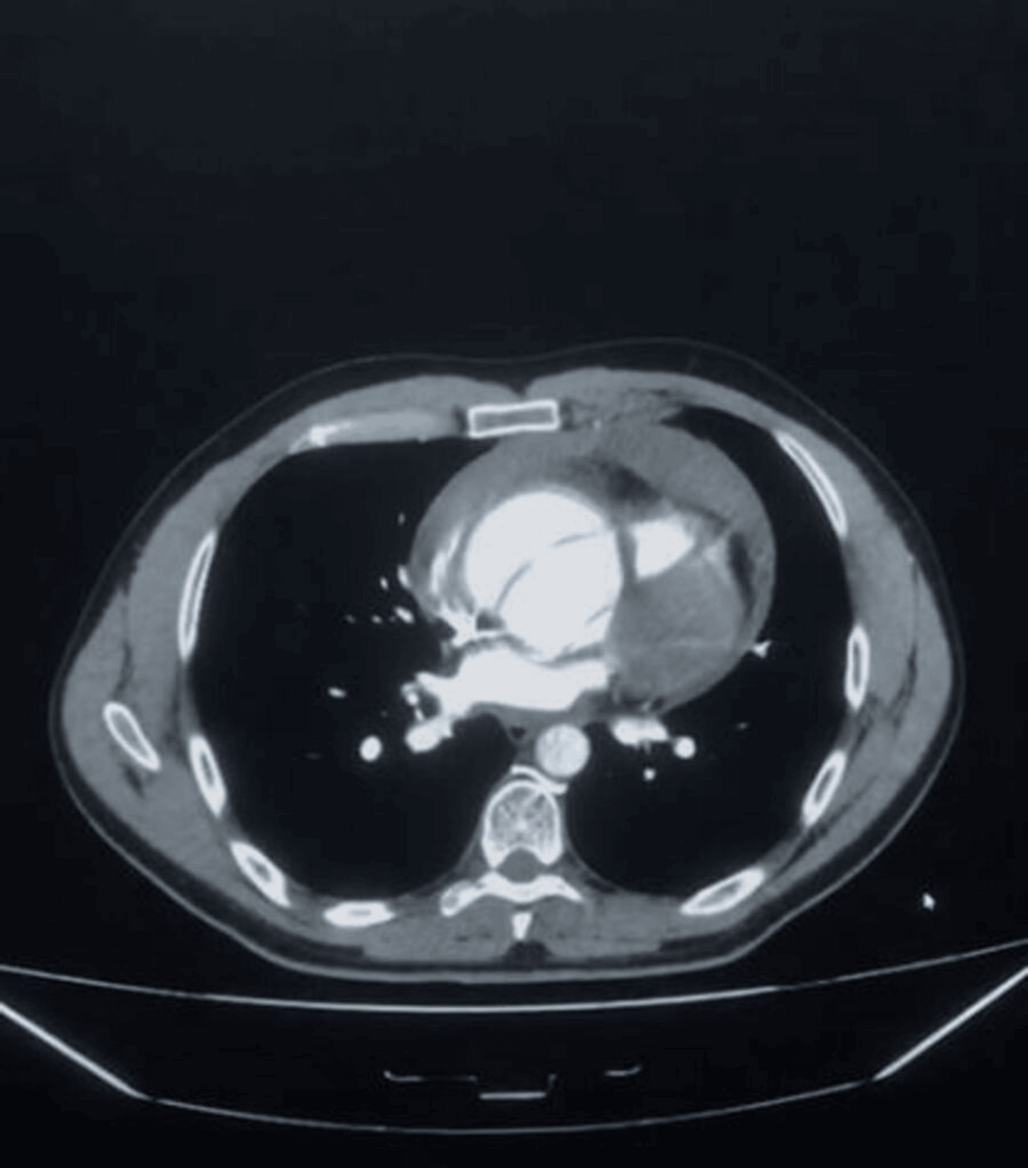
CT with contrast showing aortic aneurysm

Within 48 hours, he returned to the ED with worsened symptoms: hypotension (BP 100/60 mmHg, MAP 73 mmHg), tachycardia (HR 105 bpm), near-syncope, worsening abdominal pain, and new numbness in his legs. He appeared pale and diaphoretic, with muffled heart sounds and JVD, raising suspicion of cardiac tamponade. A CT angiogram showed an ascending aortic dissection (5.1 cm), with a dissection flap extending proximally and a significant pericardial effusion suggestive of impending tamponade. There was no descending aortic involvement or signs of visceral or renal artery compromise (Figure 1). Another image shows the aneurysm (Figure 2).

The patient was admitted to the ICU for close monitoring and blood pressure control, with preparations for emergency surgery. The next day, a Bentall procedure was performed, including resection of the dissected aortic root, placement of a 30-mm tube graft, and replacement of the aortic valve with a 25-mm mechanical valve. The pericardial effusion was evacuated, and the fused bicuspid aortic valve was addressed. Postoperatively, the patient was managed in the ICU on ventilatory support and continuous nitroprusside infusion. Echocardiography revealed good left ventricular function with moderate aortic regurgitation. He was successfully stabilized, weaned off vasopressors, and extubated without further complications, with early diagnosis and intervention proving critical in his recovery.

## Discussion

The natural history of bicuspid aortic valves (BAV) in adults shows a significant risk of progressive aortic dilatation and subsequent dissection [2]. Aortic dissection is a potentially fatal condition that demands immediate medical and surgical intervention, particularly in the case of Type A dissections, which can lead to severe complications such as cardiac tamponade or aortic rupture. Aortic dissection is typically seen in older adults, particularly those with hypertension or connective tissue disorders, often presenting in the fourth or fifth decade of life. However, patients with BAV can experience dissection much earlier, sometimes in their 20s or 30s [3]. While aortic dissection is rare in young adults, it is notably more common in those with BAV. Nienaber et al. found that approximately 30% of patients with acute aortic dissection are under 50, with some associated specifically with BAV presenting even earlier [4]. This underscores the importance of high clinical suspicion for aortic dissection in younger patients with BAV.

In such patients, dissection tends to present at a younger age, often in the fourth or fifth decade of life. Risk factors for aortic dissection include aortic aneurysms, hypertension, and connective tissue disorders. Imaging plays a critical role in diagnosing acute aortic dissection and in monitoring patients at increased risk for aortic disease. BAV-associated aneurysms are frequently associated with aortic dissection, necessitating close monitoring. BAV-associated aneurysms significantly increase the risk of aortic dissection, necessitating close monitoring. Clinicians should stratify risk in patients with BAV by considering factors such as aneurysm size, symptoms, and family history of aortic disease. Regular imaging and clinical assessments can help identify those at higher risk, allowing for timely intervention and tailored management strategies [5], [6].

Aortic dissection in younger patients is rare, particularly in those without significant predisposing factors like hypertension or connective tissue disorders. Here, the patient’s initial presentation showed atypical symptoms, including epigastric pain, lower leg numbness, and delayed recognition. Early CT imaging and high clinical suspicion were critical in diagnosing this life-threatening condition. BAV disease is clinically important because it often leads to early onset aortic stenosis and other aortic pathologies. Additionally, studies have shown that BAV is frequently linked to aortopathy, including aortic dilatation and an elevated risk of dissection, highlighting the need for routine monitoring [7].

The Bentall procedure is the definitive treatment for Type A aortic dissection with associated aortic valve disease or aneurysm [8]. Aortic dilation in BAV disease can progress over time, emphasizing the need for early detection. The 2014 European Society of Cardiology (ESC) Guidelines recommend that all patients with BAV undergo routine aortic imaging to detect complications [9]. The role of genetic mutations in the development of aortic aneurysms, such as those found in the FBXL5 gene, has been increasingly recognized in non-syndromic thoracic aortic aneurysm cases, suggesting the importance of genetic screening in at-risk populations [10]. Aortic dissection can develop in patients with undiagnosed familial BAV, highlighting the importance of early genetic screening [11].

This case highlights the importance of early recognition and prompt surgical intervention in atypical presentations of aortic dissection, as timely intervention can significantly improve survival. Bedside ultrasound (US) is crucial for the rapid identification of aortic dissection in emergency settings, where time is critical. Studies have shown that bedside US can significantly reduce the threshold of suspicion, enabling clinicians to promptly evaluate the aorta and detect abnormalities such as pericardial effusion or aortic wall irregularities. The surgical outcome was positive due to the rapid stabilization and decision for operative management. Long-term management for these patients should include routine echocardiographic assessments to monitor for aortic dilation and valvular function, as the risk of complications may persist even after successful surgery [12,13].

BAV disease is often hereditary, and individuals with this condition have an increased risk of developing aortic dilation and dissection, often without prior symptoms. Early genetic screening can facilitate the identification of at-risk family members, allowing for proactive monitoring and intervention [14]. Genetic counseling can also provide valuable insights into the inheritance patterns of the condition, empowering patients and their families to make informed decisions regarding their health [15].

## Conclusions

Dissecting aneurysm of the aorta is a highly lethal condition, often presenting with severe chest pain. This case highlights the importance of maintaining a high clinical suspicion, especially in younger patients, where diagnosis may be challenging due to the lack of classic risk factors. Timely diagnosis and intervention are critical in managing aortic dissections due to their high mortality risk. Vigilance is particularly important in younger individuals who may not present with the typical risk factors for aortic dissection.

For patients with a bicuspid aortic valve, as seen in this case, regular monitoring and early intervention are essential due to the potential for progressive aortic complications. Familial predispositions must also be considered, as undiagnosed genetic factors can increase the risk of severe outcomes like aortic dissection. Even asymptomatic patients with a bicuspid aortic valve require regular follow-up because of the risk of developing aortopathy. Aortic dilation in bicuspid aortic valve disease can progress over time, emphasizing the need for early detection. Routine aortic imaging and genetic screening are vital for identifying complications and preventing serious outcomes in these patients.
